# Modified blade: an interventional option in rigid bronchoscopy for non-resectable benign tracheal stenosis

**DOI:** 10.1186/s13019-024-02576-3

**Published:** 2024-02-08

**Authors:** Gaetana Messina, Vincenzo Di Filippo, Francesca Capasso, Maria Antonietta Puca, Beatrice Leonardi, Mario Grande, Anna Rainone, Francesco Leone, Giuseppe Vicario, Simona De Gregorio, Giuseppe Cerullo, Antonio Ponticiello, Mario Pirozzi, Stefano Farese, Alessia Zotta, Giovanni Natale, Giovanni Messina, Giovanni Vicidomini, Alfonso Fiorelli, Fortunato Ciardiello, Morena Fasano

**Affiliations:** 1 Thoracic Surgery Unit, Università Degli Studi Della Campania “Luigi Vanvitelli”, Via Pansini 5, 80131 Naples, Campania Italy; 2 Highly Specialized Medical-Surgical Department, Università Degli Studi Della Campania “Luigi Vanvitelli”, Naples, Campania Italy; 3 Oncology, Department of Precision Medicine, Università Della Campania “L. Vanvitelli”, Naples, Campania Italy; 4 Nutrition Science, University of Foggia, Foggia, Italy; 5 Pneumology Unit, Hospital of Caserta, Caserta, Italy

**Keywords:** Tracheal stenosis, Modified blade, Rigid bronchoscopy

## Abstract

**Introduction:**

Benign tracheobronchial stenosis is a abnormal tracheal lumen narrowing that may incur progressive dyspnea and life-threatening hypoxemia. There is no consensus on which patients should be treated with endoscopic or surgical method. This study investigates the outcomes of bronchoscopic dilatation in the treatment of benign tracheal stenosis using a device equipped with a blade to cut the stenotic lesions with dense fibrosis.

**Materials and methods:**

The procedure was carried out in an operating room under general anesthesia. All patients were intubated with a Rigid Bronchoscope (RB) placed just above the stenosis. Through Rigid Bronchoscopy combined modalities were used as needed: radial incisions of the mucosal stenosis with blade at the levels of 4, 8 and 12 o’clock, with back and forth movements, then the stenotic area was dilated more easily with a rigid bronchoscope. Dilatation was performed by passing the RB of increasing diameter through stenotic areas and then Balloon dilatation of increasing diameter. There were no complications during the procedure.

**Result:**

We conducted an observational, retrospective, single-centre study in the Thoracic Surgery Unit of the University of ‘Luigi Vanvitelli’ of Naples from November 2011 to September 2021. We included all consecutive patients with benign tracheal stenosis inoperable. During the study period, 113 patients were referred to our department with benign tracheal stenosis inoperable. 61 patients were treated with the blade. During the follow-up, a recurrence of the stenosis was observed in 8 patients in the first month and in 4 patients in the third month. Instead in the patients treated with the use of laser (52 patients), during the follow-up a recurrence was observed in 16 patients in the first month and in 6 patients in the third month; no patient relapsed after 6 months and after 1 year. Long term successful bronchoscopic management with blade was attained by 99% in simple and 93% in mixed stenosis and in complex type stenosis.

**Conclusion:**

Our study underlines the importance of the use of the blade in bronchoscopic treatment as a valid conservative approach in the management of patients with inoperable benign tracheal stenosis as an alternative to the use of the laser, reducing the abnormal inflammatory reaction in order to limit recurrences.

## Introduction

Benign tracheobronchial stenosis is a challenging medical condition and abnormal tracheal lumen narrowing that may incur progressive dyspnea and life-threatening hypoxemia. Tracheal stenosis (TS) can affect any level of the trachea from the cricoid cartilage to the main carina, that can impair sufficient airflow and cause severe morbidity. TS may be idiopathic or congenital but most frequently it is secondary to pathologies including malignancies, tracheal trauma, extrinsic compression or iatrogenic, inhalation burns and irradiation; however the most common causes of acquired benign tracheal stenosis are tracheostomy and endotracheal intubation [1]. The loss of regional blood flow following mucosal ischemia and ulceration produced by cuff pressure on the adjacent tracheal wall occurs within the first few hours of intubation. The healing of the damaged region can result in web-like fibrosis within 3–6 weeks; healing determines the formation of a solid fibrous scar responsible for various degrees of stenosis; concentric stenosis is the most common finding [2, 3]. During the bronchoscopic examination, the characteristics of the stenosis were assessed: “Complex stenosis” showed the presence of stenosis segments > 1 cm, malacia, cartilage involvement, and inflammation, while “Simple stenosis” showed the presence of stenosis segments < 1 cm, with involvement limited to the mucosa and absence of malacia and cartilage loss [4]. Bronchoscopic treatment is preferred for simple stenosis, while tracheal sleeve resection may be required for complex lesions [5, 6]. There is still no consensus on which patients should be treated with endoscopic or surgical method. Moreover, both endoscopic and surgical treatments involve many modifications [7]. This study aimed to investigate the outcomes bronchoscopic dilatation for treating benign tracheol stenosis using a device equipped with a blade to cut the stenotic lesions with dense fibrosis to minimize injury of adjacent normal tracheal mucosa.

### Material and method

The study was conducted in compliance with the Declaration of Helsinki and all participants signed informed consent from during preoperative communication; the protocol was approved by the Ethics Committee (32655/2021) of the University of ‘Luigi Vanvitelli’ of Naples.

**Fig. 1:**
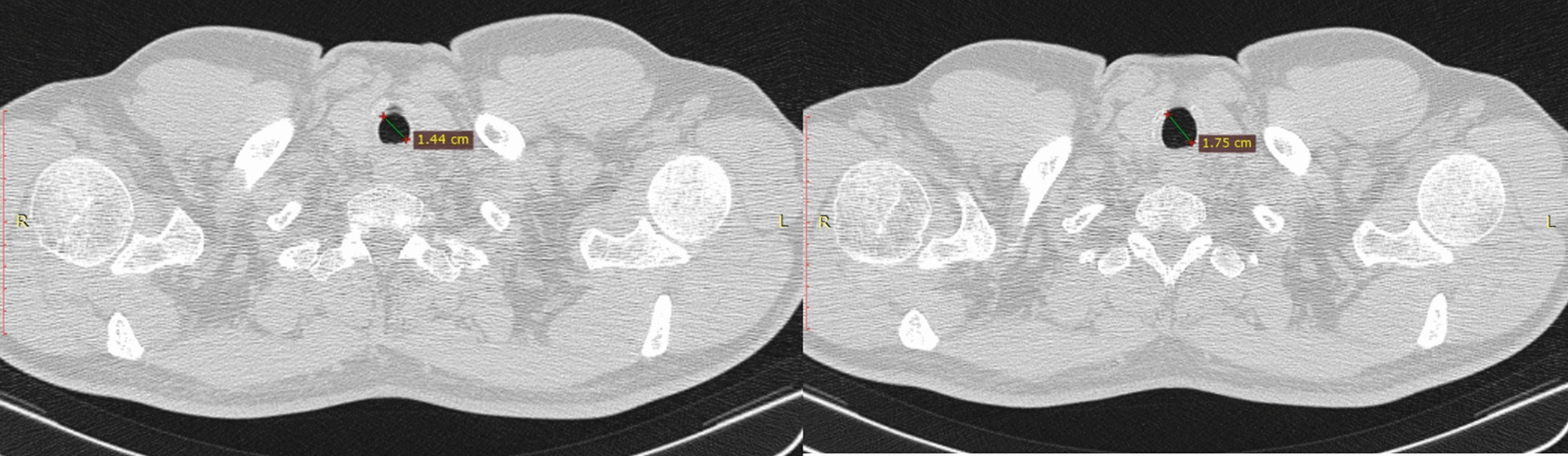
Benign tracheobronchial stenosis is a challenging medical condition and abnormal tracheal lumen narrowing

It was a retrospective study that included all consecutive patients with benign tracheal stenosis in the Thoracic Surgery Unit of the University of ‘Luigi Vanvitelli’ of Naples from November 2011 to September 2021. All patients were undergoing diagnostic preliminary flexible bronchoscopy, defining type, the localization, and severity of the tracheal stenosis. The type of stenosis was classified as simple, complex or mixed based on the length and the involvement of the tracheal wall cartilages [4] (Fig. 1). The location of stenosis was identified and classified into upper, middle, or lower-third of the trachea stenosis during flexible bronchoscopy [8]. The degree of stenosis was also recorded (0, no stenosis; 1: ≤ 25% decrease in the cross-sectional. area; 2: 26–50%; 3: 51–75%; 4: 76–90%; or 5: 91% to complete obstruction) as described in detail by Freitag et all [9, 10].

Axial computed tomography (CT) images of 1 mm thickness were used for stenosis measurement, In the localization where the lumen of the trachea is the smallest in the stenosis area was defined as stenosis size, measurements were made from the edge of the stenosis on the lumen surface to the outside edge of the trachea was defined as tissue.

The procedure was carried out in an operating room under general anesthesia [11] with pre-oxygenation with high flows nasal oxygen therapy about thirty minutes before rigid bronchoscopy, puff of lidocaine spray, using standardized intravenous techniques induction with Propofol 2 mg/Kg, Fentanyl 0.2 mg, Rocuronium 0.6 mg/Kg, Propofol in infusion in syringe pump 4 mg/Kg/hour [12]. All patients were then intubated with a rigid bronchoscope. The procedure was performed by a team of thoracic surgeons. The rigid bronchoscope was placed just above the stenosis and held firmly in place [13, 14]; through the working channel the following were introduced: optics, aspirator cannula and modified blade, flexible bronchoscope, dilatation catheter, etc. The study patients were divided into two groups: a group in which we used the blade and a group in which we used the laser to cut the fibrous stenosis. It was a retrospective study, so the two groups were not randomized. They were treated with each modality according to the availability of instruments.

**Fig. 2:**
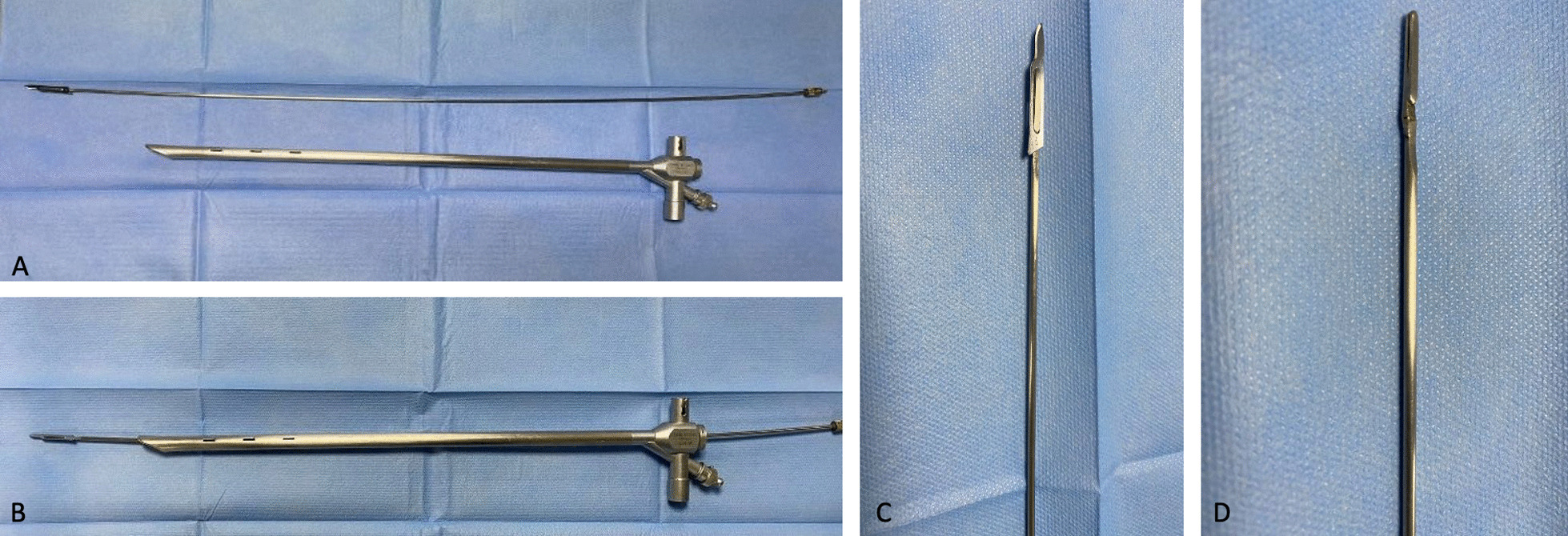
**A** a cylindrical metal handle, about 70 cm long and about 2 mm wide, sterile, in uncoated stainless steel, multipurpose, provided with a blunt distal end which acts as a handle and a proximal portion provided with a blade holder; **B** through the working channel the following were introduced: optics, aspirator and endoscopic forceps and new device: ‘Modified Blade’; **C**, **D** Proximal portion provided with a blade holder n. 3 in stainless steel, in correspondence with which a blade No. 11 is inserted, disposable, extremely sharp and resistant, straight, triangular in shape, with cutting tip, flat back and parallel to the handle, used for small incisions and precision cuts

**Fig. 3:**
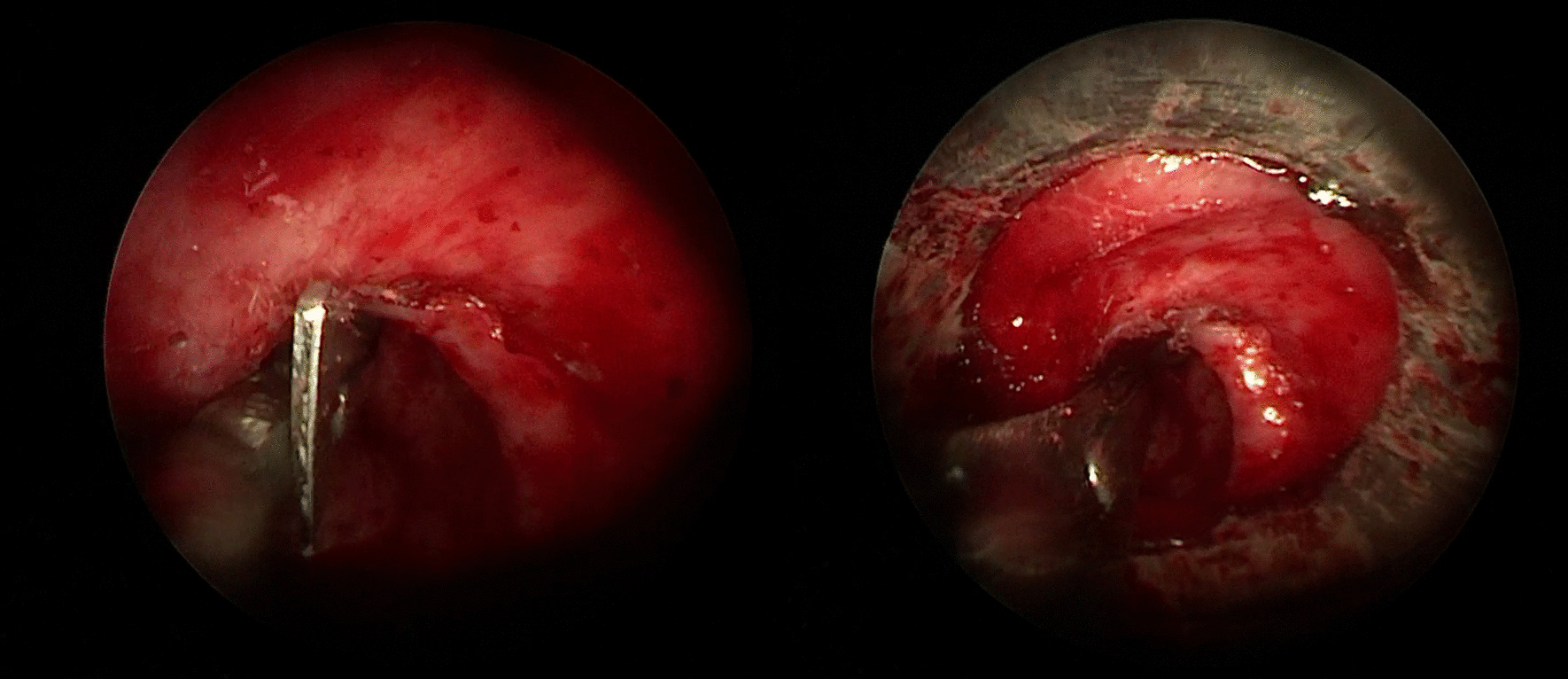
Radial incisions of the mucosal stenosis with blade were sectioned at the levels of 4, 8 and 12 o’clock, with back and forth movements, delicate, light and extremely precise, then the stenotic area was dilated more easily with a rigid bronchoscope

**Fig. 4:**
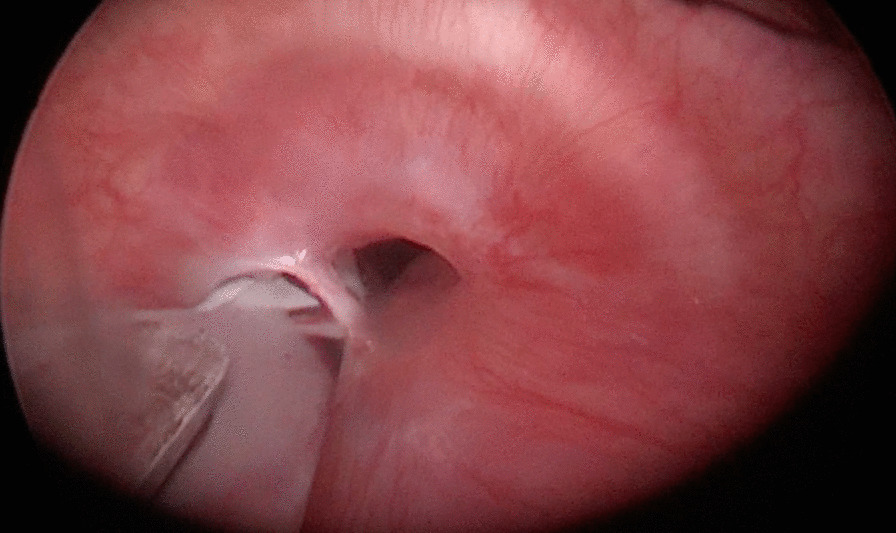
Radial incisions of the mucosal stenosis with blade were sectioned at the levels of 12 o’clock, with back and forth movements, delicate, light and extremely precise, then the stenotic area was dilated more easily with a rigid bronchoscope

A new device (Fig. 2) designed by us was introduced through the rigid bronchoscope. It is a cylindrical metal handle, about 70 cm long and about 2 mm wide, sterile, in uncoated stainless steel, multipurpose, provided with a blunt proximal end which acts as a handle and a distal portion (Fig. 20 provided with a blade holder n. 3 in stainless steel, in which a blade No. 11 is inserted, disposable, extremely sharp and resistant, straight, triangular in shape, with cutting tip, flat back and parallel to the handle, used for small incisions and precision cuts (Fig. 2). The modified blade has such a caliber that it can be used in bronchoscopes of any size. Under vision the new device was inserted into the operating canal and delicately advanced until the stenosis, orienting the cutting part upwards. We advance the modified blade at the same time as the optics so as to visualize the direction of the blade and the depth of the cut in real time, avoiding damaging the intact tracheal wall and any complications. Through Rigid Bronchoscopy combined modalities were used as needed: radial incisions of the mucosal stenosis with blade were sectioned at the levels of 4, 8 and 12 o’clock, with back and forth movements, delicate, light and extremely precise, advancing the optics synchronously with respect to the blade moments in order to visualize the distal part of the blade, performing the entire procedure under direct vision in real time. Once the points of greatest strength of the stenosis have been reduced, the stenotic area was dilated more easily with a rigid bronchoscope (Figs. 3, 4). Dilatation was performed by passing the RB of increasing diameter through stenotic areas and then Balloon dilatation of increasing diameter [15], thus permitting a satisfactory dilatation of the tracheal stenosis [16]. If the passage of the increased caliber rigid bronchoscope is obstructed, then a further incision is made. Then Balloon dilatation of increasing diameter, thus permitting a satisfactory dilatation of the tracheal stenosis. The balloon (15–16.5–18 mm.) was inflated to a predetermined pressure corresponding to the desired diameter, applying controlled radial pressure to the stricture. Balloon dilatation exerts a radial expansible force in the stenotic area and distributes this force over the entire circumference of the stenosis, avoiding rupture at any point. A diameter of 15 mm can be achieved with a pressure of 4.5 atm, and a diameter of 18 mm with 7 atm (at a length of 5.5 cm).according to the patient’s oxygen reserves and saturation during inflation. At the end of the session, we removed the catheter to allow ventilation and to control the result of satisfactory dilatation of the tracheal stenosis.

There were only minimal complications: minimal bleeding treatment with compression exerted by the same bronchoscope. In the other group, the stenosis was treated using Thulep Laser with high pulse emission frequencies (up to 1000 Hz); the dilatation was finally carried out by passing RB of increasing diameter through stenotic areas.

We decided not to use the stent after the first treatment since both the complex and simple stenoses had been well dilated. We positioned the stent in the patients who relapsed.

Repeated procedures were performed in patients that relapsed. They were treated with dilatation and stent 8 patients in the first month and 4 patient in the third month. No one underwent surgical resection, because of their poor general conditions.

Upon awakening, all patients maintained spontaneous breathing with saturation values of about 94–95% (93 ± 1.34%).

All the patients were followed with fiberoptic bronchoscopy at 1st, 3rd, 6th, 9th, 12th, 18th, and 24th month after the initial treatment [16, 17].

### Statistic analysis

In the group of patients treated with the blade during the follow-up the stenosis relapsed in the first month in 8 patients and in the third month in 4 patients, while in those ones treated with the use of the Laser during the follow-up, in 16 of them relapsed in the first month and in 6 patients in the third month, no patient recurred after six months and after a year. Long term successful bronchoscopic management with blade was attained by 99% in simple and 93% in mixed stenosis and in complex type stenosis.

**Fig. 5:**
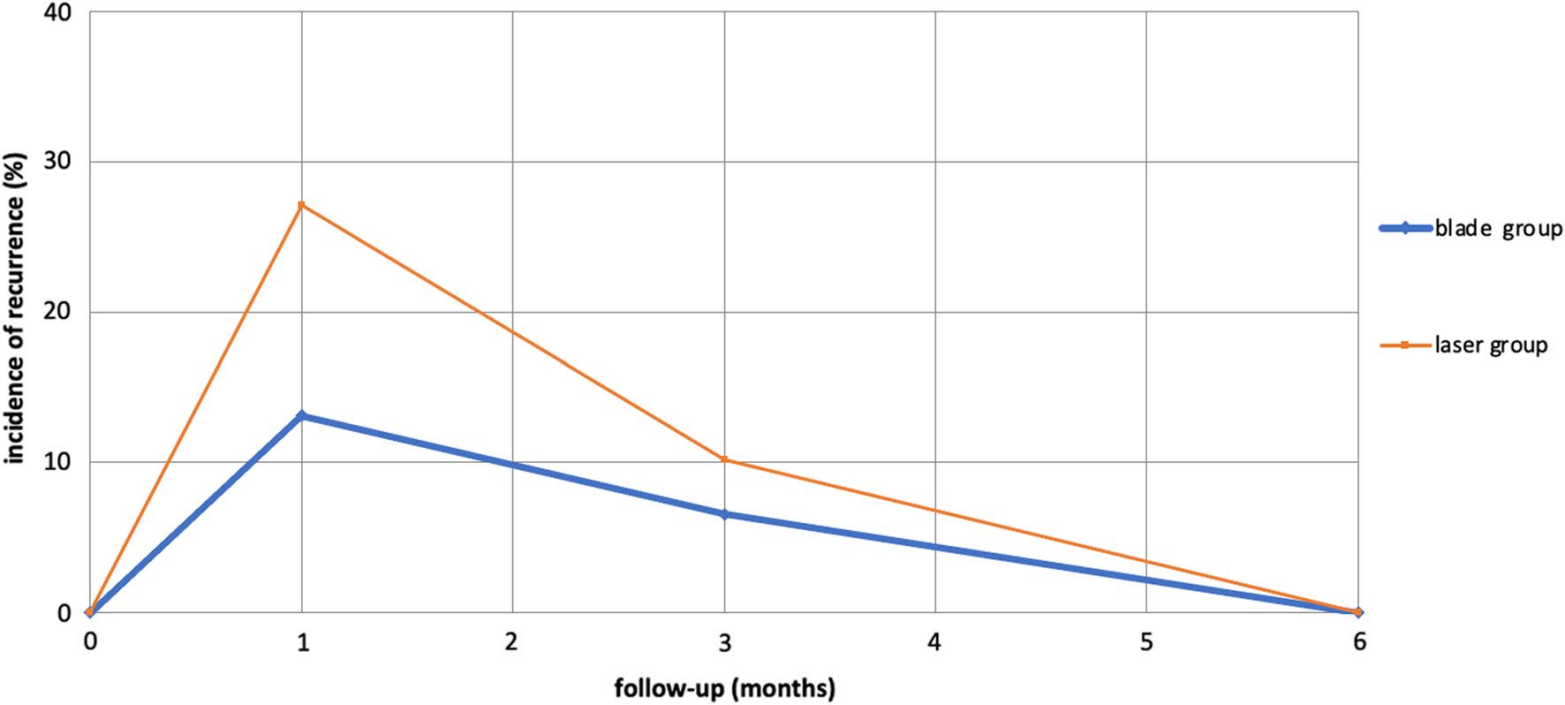
Recurrences after treatment in rigid bronchoscopy

The Pearson correlation test was used to evaluate the correlation between the use of blade and number of recurrences after treatment in rigid bronchoscopy. [Fig. 5].

A *P*-value < 0.05 was considered statistically significant. MedCalc statistical software (version 12.3; Broekstraat 52,9030 Mariakerke, Belgium) was used for the analysis (Table 1).

**Table 1:** The stenosis relapsed during follow-up

Recurrence (months)	Blade (n = 61)	Laser (n = 52)	*P* value
	n (%)	n (%)	
1st	8 (13)	16 (27)	0.16
3rd	4 (7)	6 (10)	0.68
6th	–	–	
12th	–	–	
24th	–	–	
Operating time (min)	18 ± 2.3	35 ± 3.5	< 0.001

## Result

We included all consecutive patients with inoperable benign tracheal stenosis. During the study period, 113 patients were referred to our department with benign tracheal stenosis at initial presentation and were enrolled in the study. The stenosis affected the superior third of the trachea in 61 cases (54%), the middle third in 29 cases (26%), the lower third in 23 cases (20%). 68 patients had post intubation stenosis and 45 patients had post tracheostomy stenosis. The mean age of the study population was 69 years (65 ± 5.83 years); 48 were women and 65 were men. The causes of prolonged intubation were as follows: 45 patients with myocardial infarction, 38 patients with respiratory failure, 11 patients with covid pneumonia, 2 patients with post-operative complications from colon resection, 9 strokes, 8 patients for trauma. Regarding the duration of intubation, 60% of Tracheal Stenosis Post Intubation (PITS) cases were intubated for a period of less than 7 days. All patients complained of dyspnea and easy fatigability, 25 patient tirage, 31 patients respiratory failure, 7 patients exertional dyspnea, 18 patients cornace. All patients were judged inoperable due to serious comorbidities: severe heart failure, coagulation disorders, severe respiratory failure. (The clinical characteristics of the patients are presented in Table 2). Amongst the studied population, 29 patients (26%) had complex stenosis, presence of stenosis segments > 1 cm, accompanied by cartilage involvement, malacia, and inflammation; while 76 patients (67%) had simple < 1 cm, with involvement limited to the mucosa and absence of malacia and cartilage loss and 8 patients (7%) had mixed stenosis. The degree of residual lumen and the length of a simple tracheal stenosis were 25.3% and 0.91 cm (range, 0–1 cm), respectively. The degree of residual lumen and lengths of the complex tracheal stenosis were 18.5% and 2.8 cm (range, 1–3 cm), respectively. In total, 201 rigid and 452 flexible bronchoscopic procedures were performed in patients with complex and simple stenoses. 61 patients were underwent radial incisions of the mucosal stenosis with blade No. 11 sectioned at the levels of 4, 8 and 12 o’clock, while 52 patients the treatment of the stenosis was carried out with methods combined with the use of the laser and and dilatation by passing the RB of increasing diameter through stenotic areas. The dilatation of the stenosis in the patients in which the blade was used was easier and faster than in those ones in which the laser was used (23 min vs. 35 min); Then in all patients dilatation was completed by balloon dilatation of increasing diameter. Post-procedural complications were relatively minor and manageable: no severe intra-procedural complications nor bleeding neither respiratory failure nor pneumomediastinum or nor mortality were observed. Surveillance bronchoscopy was carried out routinely at regular intervals (1, 3, 6, 12, 18, 24 months). In the group of patients treated with the blade during follow-up the stenosis relapsed in the first month in 8 patients and in the third month in 4 patients, while in the patients treated with the use of the Laser during follow-up in 16 patients relapsed in the first month and in 6 patients in the third month, no patient recurred after six months and after a year. Long term successful bronchoscopic management with blade was attained by 99% in simple and 93% in mixed stenosis and in complex type stenosis. We considered as successful the bronchoscopic treatment that did not relapse and did not require any additional intervention or the positioning of a stent.

**Table 2:** The clinical characteristics of the patients

Variables	All patients (n = 113)	PITS (n = 68)	PTTS (n = 45)	*P* value
Age, years (median)	65 ± 5.83	65.5 ± 5.33	65 ± 5.13	0.66
Sex (male), n (%)	65 (55)	37 (54)	28 (62)	0.46
Myocardial infarction, n (%)	45 (38)	26 (38)	19 (42)	0.67
Respiratory failure	38 (32)	23 (34)	15 (33)	0.91
Covid pneumonia	11 (9)	7 (10)	4 (9)	0.86
Colon resection	2 (2)	2 (3)	0 (0)	0.24
Stroke	9 (8)	5 (7)	4 (9)	0.84
Trauma	8 (7)	3 (4)	5 (11)	0.15
Dyspnea	113	68	45	1
Tirage	25 (21)	15 (22)	10 (22)	1
Exertional dyspnea	7 (6)	4 (6)	3 (7)	0.83
Cornage	18 (15)	11 (16)	7 (16)	1
Complex stenosis	29 (24)	17 (25)	12 (27)	0.81
Simple stenosis	76 (64)	47 (69)	29 (64)	0.58
Mixed stenosis	5 (4)	2 (3)	3 (7)	0.32

## Discussion

Currently there is no definitive consensus on the management of tracheal stenosis. Interventional bronchoscopy includes laser, stenting and mechanical dilatation, the application of which in tracheal surgery has increased in recent years [18, 19]. Literature data show that only simple stenosis, including cases of granuloma or thin web-like stricture, can be permanently removed by dilatation, laser treatment, or laser-assisted mechanical dilatation; while, the benefit in more complex lesions is temporary, with frequent recurrences [20, 21], with the need to repeat procedures and the risk of the widening diseased segment [22]. Today, tracheal resection of the stenotic part of the airway followed by end-to-end anastomosis is the gold standard for the management of benign TS [23–25]. Respiratory, neurological, and cardiovascular comorbidities are major barriers to surgery. However, optimal management is not universally standardized, and therefore remains controversial. In cases not amenable to surgery, bronchoscopic management of tracheal stenosis is an alternative to surgery [26], and endoluminal treatment can play an important role in the management of both simple and complex cases of tracheal stenosis [27]. As reported by previous studies, excessively long benign tracheal stenosis (approximately 50% or more of the entire tracheal length) [28], as well as the consequences of head injury, respiratory failure, neurosurgery, cardiovascular disease, or other debilitating diseases [29], represent absolute contraindications. To surgical management. Galluccio et al. reported 69% success for complex tracheal stenosis treated primarily with laser resection [30]. Similarly, Cavaliere et al. indicated good results in the treatment of both simple and complex tracheal strictures.

Therefore, in cases of surgical ineligibility, endoscopic treatment may potentially be a good choice even for complex stenosis, at least as a bridge to operative treatment. Argon plasma coagulation was the most commonly used bronchoscopic treatment for complex stenoses, followed by diode laser treatment and cryotherapy.

Surgically, the laser can be used to “cut” tissue, and indeed the CO2 laser is frequently used for the treatment of subglottic tracheal stenosis. The CO2 laser is also a “line of sight” laser, and the laser pulse will travel until it is absorbed by tissue or another protective material (such as a saline-soaked sponge). However, the use of laser resection has been reported to be associated with a higher recurrence rate in cases of complex tracheal strictures. At the present time, almost all medical application of lasers relates to thermal interaction mechanisms—the conversion of optical radiation energy into heat and the resulting coagulation or vaporization of the heated tissue.

The Argon -laser is suited chiefly for coagulation of blood vessels. Because of the strong absorption of its blue and green radiation in hemoglobin. As a consequence of its relative low absorption and scattering the Nd:YAG-laser beam penetrates deeper into the tissue, and therefore it presents itself for deep coagulation of tissue. This type of laser also may be used for cutting, mainly in such cases where a thick zone of necrosis is desirable, for instance for cutting tissue rich in blood, because the induced shock wave causes destruction of tissue [31, 32] Laser treatment is therefore an effective modality for the treatment of obstructive airway lesions [33]. However, the operator must be careful to avoid extensive damage to the bronchial wall, leading to further cartilaginous damage and fibrosis and subsequent recurrence of the luminal stenosis [34, 35].

Therefore, the laser can lead to excessive and aberrant wound healing, due to deregulation of one of the three phases of healing, leading to a state of chronic localized inflammation, accumulation of fibroblasts [36] and connective tissue and subsequent possibility of recurrence of the stenosis. The use of the blade instead induces a more physiological wound healing process through the phases of hemostasis, inflammation, proliferation, and remodeling, without excessive wound healing. Therefore, in our experience we have observed that the use of the blade in benign tracheal stenosis reduces recurrences.

Our experiences suggest that the blade is an ideal instrument to establish the airway. The blade is inserted directly into the working channel, and its tip is directly visualized by rigid bronchoscopy. Thus, its direction and location can be confirmed within the tracheal lumen. To ensure the blade is directed towards the site of stenosis, the rigid bronchoscope is used as directional guidance for the blade to cut the strongest points of the stenosis. Therefore, we can prevent the complications and damage to the adjacent tissues. In our study, all patients with tracheal stenosis achieved recanalization using a blade, it is a promising and effective treatment for complete tracheal stenosis. The novel approach provides a basis for further therapies such as dilatation and gives the patients an opportunity for restored airway patency, which can improve their quality of life. It is less invasive and more beneficial to patients compared to open surgery and conventional interventions.

## Conclusion

Bronchoscopic treatment is not only a palliative alternative to surgery, but should be considered as a viable option for selected cases of complex tracheal stenosis. Our study underlines the importance of the use of the blade in bronchoscopic treatment as a valid conservative approach in the management of patients with tracheal stenosis as an alternative to the use of the laser, reducing the abnormal inflammatory reaction in order to limit recurrences as much as possible. However, further studies are needed to corroborate our data.

## Data Availability

Data is contained within the article.
